# A systematic literature review of spinal brace/orthosis treatment for adults with scoliosis between 1967 and 2018: clinical outcomes and harms data

**DOI:** 10.1186/s12891-020-3095-x

**Published:** 2020-02-08

**Authors:** Jeb McAviney, Johanna Mee, Azharuddin Fazalbhoy, Juan Du Plessis, Benjamin T. Brown

**Affiliations:** 1 Sydney Scoliosis Clinic, Kirk Place, Level 5, Suite 5.08, 15 Kensington St, Kogarah, NSW 2217 Australia; 2 Melbourne Scoliosis Clinic, Ground Floor, Suite 3, 492 St Kilda Road, Melbourne, VIC 3004 Australia; 3 School of Health and Biomedical Sciences, RMIT University, Bundoora, VIC 3083 Australia; 4 Department of Chiropractic, Macquarie University, North Ryde, NSW 2109 Australia

**Keywords:** Braces, Adult, Scoliosis, Therapeutics, Pain

## Abstract

**Background:**

There is a paucity of literature regarding the conservative management of adult scoliosis. The authors review and summarize the literature from 1967 to 2018 on the clinical outcomes of spinal brace/orthosis use in this subgroup of the population.

**Methods:**

CINAHL, Embase, CENTRAL, PubMed and PEDro were searched from database inception to the 30th of October, 2018. A combination of medical subject heading terms and keywords pertaining to three core concepts (adult, scoliosis, and braces/orthoses) were used in the search. Studies were included if A) clinical outcomes were collected from B) participants ≥18 years C) receiving spinal brace/orthosis treatment for D) primary degenerative (de novo*)* scoliosis or progressive idiopathic scoliosis. A step-wise screening process was employed which involved a title and abstract screen for relevancy followed by a full text eligibility appraisal by two authors. Data were extracted, and a risk of bias assessment was performed on the included cohort studies using the Newcastle-Ottawa Scale. Given the overall level and quality of the available evidence, conclusions were drawn based on a qualitative summary of the evidence.

**Results:**

Ten studies (four case reports and six cohort studies) were included which detailed the clinical outcomes of soft (2 studies) or rigid bracing (8 studies), used as a standalone therapy or in combination with physiotherapy/rehabilitation, in 339 adults with various types of scoliosis. Most studies included female participants only. Commonly reported outcomes were pain (7 studies), function (3 studies) and Cobb angles (3 studies), with follow-up times ranging from 2 days to 17 years. Brace wear prescriptions ranged from 2 to 23 h per day, and there was mixed brace-compliance reported. Most studies reported modest or significant reduction in pain and improvement in function at follow-up. There were mixed findings with regards to Cobb angle changes in response to bracing. Participants from one study noted discomfort associated with bracing. Each of the six cohort studies demonstrated a high risk of bias.

**Conclusion:**

There is evidence to suggest that spinal brace/orthosis treatment may have a positive short – medium term influence on pain and function in adults with either progressive primary (de novo) degenerative scoliosis or progressive idiopathic scoliosis. At this point in time the evidence is of low quality and has been focused primarily on female patients with thoracolumbar and lumbar curves. More granular statements regarding the efficacy of different brace types or manufacturers, or the effect of this therapy on different curve types cannot be determined based on the current literature. Properly constructed prospective trials are required to better understand the efficacy of bracing in adult scoliosis.

## Background

Scoliosis represents a deviation of the spine in the coronal plane with associated vertebral rotation [1]. Whilst there are many different types of scoliosis that may manifest at various points across the lifespan, several variants have been identified that are specific to adult populations - see Aebi [2] for a full review and classification. The most common types of adult scoliosis include primary de novo degenerative scoliosis and progressive idiopathic scoliosis [2]. Primary degenerative scoliosis represents a new (de novo*)* curve that develops in patients with no prior history of scoliosis, and typically affects the lumbar or thoroacolumbar spine. The prevalence of the primary degenerative scoliosis has been reported to be as high as 68% in individuals aged 60–90 years [3]. Progressive idiopathic scoliosis represents curve progression and spinal degeneration in adults with pre-existing idiopathic curves [2].

It has been proposed that changes in the structure, function, and physiological alignment of the spine lead to asymmetrical loading which provokes further degenerative change, accelerated curve progression and postural collapse [4]. Depending upon the timing of presentation it can be challenging to differentiate between the various types of adult scoliosis, however most patients will present with back pain accompanied by some form of progressive postural deformity [5]. In the more severe cases, patients may experience lumbar radiculopathy, myelopathy and/or intermittent neurogenic claudication due to the advanced nature of degenerative changes [6, 7] such as asymmetrical disc degeneration, spondylosis/facet incompetence and hypertrophy and calcification of the ligamentum flavum. Foraminal/lateral-recess/central stenosis and/or neural stretching or tethering can also be observed in such cases [7].

Managing the progressive nature of this condition whilst preserving quality of life can present a unique challenge. Adult patients with scoliosis are generally encouraged to explore conservative treatment options prior to undergoing surgical intervention [8]. However, there is limited evidence regarding the effectiveness of conservative treatment [9]. Bridwell et al [10] investigated the impact on quality of life (QOL) of surgical and non-surgical treatments on 160 symptomatic adults with lumbar scoliosis using a prospective observational cohort study design. The non-surgical (75 participants) treatments included observation (21%), medication (26%), medication combined with physical therapy and/or injection techniques (40%), and other treatment without medications (13%). The authors found that surgical treatment of adults with scoliosis resulted in significant improvement in QOL after 2 years, whereas the non-surgical treatments had no significant effect. Interpretation of the findings from the non-surgical group were however hampered by substantial loss to follow-up (45%).

There is a growing body of evidence regarding the influence of physiotherapeutic scoliosis specific exercise for the treatment of adolescent idiopathic scoliosis patients. However, research into the efficacy of this type of treatment for adult scoliosis patients is in its infancy. This is evidenced by a recent systematic literature review performed by Alanazi et al [11] that looked into the effects of stabilization exercises on back pain, disability and quality of life in adults with scoliosis. A comprehensive search of the available literature revealed only one randomised parallel-group, superiority-controlled trial [12] that fulfilled the author’s eligibility criteria for the review. The authors of this single randomised controlled trial sought to investigate the effects of motor and cognitive rehabilitation on disability in 130 adults with idiopathic scoliosis (low-moderate curves [< 35° Cobb-Lippman]). The intervention consisted of 20 weeks of active self-correction exercises that were reinforced with strengthening exercises and challenged with task-oriented activities and other postural perturbations. In addition, participants also received cognitive behavioural therapy and ergonomic advice. The authors of the study found significant improvements in pain, disability and QOL scores that were superior to general physiotherapy treatment. As is the case for all trials involving complex exercise interventions, blinding was not possible. Consequently, Alanazi et al assessed this trial as having a high risk of bias and called for further experimental research in this area.

There is good evidence to support the use of bracing for adolescent idiopathic scoliosis (AIS) [13]. In contrast, there is a paucity of literature regarding this type of treatment for adult patients. In adults, the thoracolumbosacral (TLSO) or lumbosacral orthoses (LSO) are comparable in appearance and utilize similar materials to those used in pediatric populations, however the purpose and proposed mechanism of action is different. Instead of trying to modulate spinal growth, as is the case in adolescent patients, the primary aim of adult bracing is to apply external forces to the spine/trunk to temporarily improve physiological spinal alignment. Adult spines are stiffer than adolescent spines and therefore less responsive to external corrective forces, so this type of therapy aims at moving a patient’s spine/trunk into the best possible physiological alignment with the intention of relieving symptoms that accompany postural deviation/collapse e.g. pain.

A recent report from the World Health Organisation suggests that by 2050, the proportion of the world’s population aged > 60 years will nearly double [14], which will likely increase the proportion of adults seeking care for adult scoliosis. It is therefore prudent to understand the efficacy of both non-surgical and surgical treatments. The aim of this study was to systematically review and summarise the existing literature from 1967 to 2018 regarding the efficacy of spinal braces/orthoses for improving clinical outcomes in adults with scoliosis.

## Methods

A search was performed in CINAHL complete (EBSCOhost), Cochrane Central register of Controlled Trials, PubMed, Embase (OVID), and Physiotherapy Evidence Database (PEDro) from inception to the 30th of October, 2018 in each database. The authors sought to retrieve all study types investigating the clinical outcomes of spinal brace/orthosis treatment for adults with scoliosis. A combination of medical subject heading terms and keywords pertaining to three core concepts (adult, scoliosis, and braces/orthoses) were used in the search. A scoping review was performed to obtain a list of brace/orthosis types/manufacturers that could be included in the search string for the purposes of increasing the sensitivity of the search. An example of the search string used for the PubMed database is detailed in theAppendix. Studies were eligible for inclusion in the review if they A) collected clinical outcomes from B) participants ≥18 years of age C) who were receiving soft/rigid/super-rigid spinal brace/orthosis treatment for D) degenerative de novo scoliosis or progressive idiopathic scoliosis in adult life and E) were peer-reviewed and published in full-text in English. A scoping review was performed to assess the state of the literature on the spinal brace/orthosis treatment for adults with scoliosis. Due to the general paucity of information on the topic revealed by the scoping review, the authors decided to omit inclusion criteria relating to specific clinical outcomes and study types in order to maximise the search results. Studies that assessed post-operative bracing/casting, or studies that braced participants with types of scoliosis other than degenerative de novo scoliosis or progressive idiopathic scoliosis were excluded.

Search results were imported into the *Endnote* bibliography management software and then duplicates were removed. A title and abstract screen was conducted separately by two authors to remove all clearly irrelevant studies. Full text copies of the remaining articles were then obtained, and the eligibility criteria were applied separately by two authors. Any disagreements were resolved by consensus. Forward (using the citations feature in the *Scopus* database) and reverse citation (bibliography screen) tracking based on the list of eligible articles was employed to identify any studies that weren’t picked up by the primary search strategy. A manual search was also performed on the Society on Scoliosis Orthopaedic and Rehabilitation Treatment (SOSORT) website [15] for proceedings from each of the annual conferences from 2004 to 2018.

Data from eligible articles were extracted independently by two authors using a data extraction template created by the research team that was piloted on several studies prior to use. The review authors sought to capture data on: study design; participant characteristics, working diagnosis; co-morbid illness; brace characteristics; proposed mechanism of action; average hours of brace-wear; brace-compliance; additional treatment received; primary and secondary outcomes; information on follow-ups (frequency and timing); study findings; and information on harms/adverse events. A risk of bias assessment of all cohort studies was performed independently by two authors using the Newcastle-Ottawa Scale (NOS) [16], and a quality rating (*unclear, high, moderate, or low*) provided for each study based on recommendations from the Agency for Healthcare Quality and Research [17]. Case studies are known to be biased and therefore a high risk of bias was automatically assigned to these study types.

After performing a scoping review, it became clear that there would likely be no systematic reviews or randomized controlled trials retrieved on the efficacy of spinal brace/orthosis treatment for adults with scoliosis. It was anticipated that this would prohibit the use of meta-analytical techniques for determining effects sizes, or the use of robust frameworks e.g. GRADE, for summarizing the research findings. The researchers instead opted for a simple qualitative summary of the research findings regarding each outcome or adverse event reported based on the balance of evidence for each outcome. Given the small quantity of literature on this topic this method should be considered suitably transparent. No additional analyses were planned. This report was prepared using the Preferred Reporting Items for Systematic Reviews and Meta-Analyses (PRISMA) document [18].

## Results

**Fig. 1:**
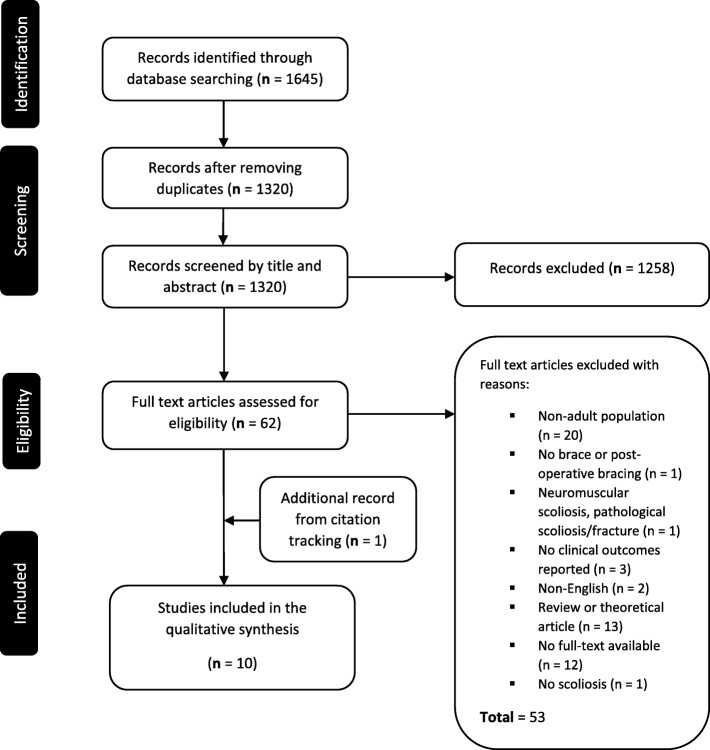
Flowchart of the search and selection process

There were 1645 records identified by the primary search strategy. Of these, 325 were flagged as duplicates and were subsequently removed. The remaining 1320 publications were then screened by title and abstract. Sixty-one publications were deemed suitable for full-text appraisal. The full-text copies of these publications were obtained, and the eligibility criteria were applied independently by two authors. There were nine eligible studies. Forward and reverse citation-tracking based on the nine eligible publications returned a further 1205 records. These records were screened by title and abstract which revealed one additional study [19]. An analysis of the proceedings from the SOSORT scientific meetings highlighted seven abstracts/oral presentations discussing the effects of bracing in adult populations. Four of these abstracts could be linked to studies that had been written up and already included in the review [20, 21]. The remaining three abstracts focused on the effects of the *SpineCor* brace in adults [22–24]. There was however insufficient detail provided in these abstracts to allow for inclusion in the analysis. For a description of the search and selection process please see Fig. 1.

Ten studies (four case reports [6, 25–27] and six cohort studies [two retrospective [19, 28] and four prospective [20, 21, 29, 30] were included which detailed the clinical outcome of soft (two) [21, 26] or rigid eight) [6, 19, 20, 25, 27–30] bracing, used as a standalone therapy [6, 21, 26, 28–30] or in combination with casting and /or physiotherapy/rehabilitation [19, 20, 25, 27], in 339 participants in total. There were six different brands of spinal brace/orthosis represented in this review (*Physiologic* [6, 29, 30], *Lyon* [19, 20], *SBrace L* [27], *Gensingen* [25], *Vesinet* [28] and the *Peak Scoliosis Brace* [21] (Table 1).

**Table 1:** Summary of the Eligible Studies

Author (Year)	Methods	Participants	Measurement	Findings
Weiss et al (2006) [6]	*Design:* Case report *Brace Type:* Lordosing spinal orthosis *Trade name:* Physiologic *Material:* Polyethylene *Proposed MOA:* Sagittal re-alignment brace that has a lordosing action in the lumbar spine *Additional treatment:* NA *Region:* Germany	*Sample size:* 1 *Age:* 47 *Sex*: 1 Female *Diagnosis:* Degenerative *Scoliosis Parameters:* 55° Lumbar scoliosis *Co-morbid Illness:* Grade II spinal claudication *Prescribed Brace Wear:* NA	*Primary outcome:* VPRS *Secondary outcome*: Walking distance *Number of F/U points*: 3 *Length of F/U:* T1 = 2 days T2 = 10 days T3 = 8 weeks	*Main finding:* Reduction of 1 point on the VPRS after 10 days that remained stable at 8 weeks *Additional findings:* Increase in walking distance from 800 to 8000 at T1, then 8000 to 12,000 at T2. Improvements maintained at T3. *Statistical significance:* NA *Brace compliance:* NA *Harms associated with bracing:* NA
Weiss et al (2006) [29]	*Design:* Prospective cohort study *Brace type:* Lordosing spinal orthosis *Trade name:* Physiologic *Material:* Polyethylene *Proposed MOA:* Sagittal re-alignment brace that has a lordosing action in the lumbar spine *Additional treatment:* NA *Region:* Germany	*Sample size:* 29 *Age:* 41 years (SD = 21) *Sex:* 29 Females *Diagnosis:* Scoliosis *Scoliosis Parameters:* 37° (SD = 22) *Co-morbid Illness:* NA *Prescribed Brace Wear:* Minimum prescription of 8 h per day for 7.5 months (SD = 5.6)	*Primary outcome*: Roland Morris verbal rating scale (0–5 point scale) *Secondary outcome:* NA *Number of F/U:* 2 *Length of F/U*: T1 = ≤1 week T2 = > 6 months (Avg = 7.5 months)	*Main finding:* A significant reduction in Roland Morris VRS between T0 and T1. Smaller but non-significant reduction in scores between T0 and T2 *Additional findings:* NA *Statistical significance:* Wilcoxon Test (*p* < 0.001) *Brace compliance:* Poor. Compliance lost in the majority of patients at the time of the follow-up period. Only 7 patients reported wearing the brace for > 4 h at the T2 *Harms associated with bracing:* NA
Weiss et al (2009) [30]	*Design:* Prospective cohort study *Brace type:* Lordosing spinal orthosis *Trade name:* Physiologic *Material:* Polyethylene *Proposed MOA:* Sagittal re-alignment brace that has a lordosing action in the lumbar spine *Additional treatment:* NA *Region:* Germany	*Sample size:* 56 *Age:* NA *Sex:* Males and females *Diagnosis:* Idiopathic = 48, congenital = 2, de novo degenerative = 3, other = 3 with chronic lower back pain *Scoliosis Parameters:* 41° (Range = 10–91) *Co-morbid Illness:* NA *Prescribed Brace Wear:* 20 h per day for 6 months then on a case-by-case basis after that point	*Primary outcome:* Roland Morris verbal rating scale (0–5 point scale) *Secondary outcome:* progression to surgery for chronic lower back pain *Number of F/U:* 2 *Length of F/U:* T1 = ≤1 week T2 = average of 18 months	*Main finding:* Reduction of Roland Morris scores of 3.3 to 2.7 at T1, then 2.7 to 2.0 at T2 *Additional findings:* 21 patients were able to completely remove the brace after 6 months due to the alleviation of pain *Statistical significance:* Wilcoxon Test (*p* < 0.05) *Brace compliance:* NA *Harms associated with bracing:* NA
De Mauroy et al (2011) [19]	*Design:* Retrospective cohort study *Brace type:* Rigid polyethylene bi-valve overlapped lumbar brace *Trade name:* Lyon brace *Material:* High density polyethylene *Proposed MOA:* Rebalancing of the spine in the frontal and sagittal plane *Additional treatment:* plaster cast (3 weeks), physiotherapy (3 weeks) *Region:* France	*Sample size:* 33 *Age:* 59.9 (SD = 10.8) [Range 33–77] *Sex:* 30 females, 3 males *Diagnosis:* Adult scoliosis (lumbar or thoracolumbar) *Scoliosis Parameters:* 37° SD = 18 (Range 10–75) *Co-morbid Illness:* NA *Prescribed Brace Wear:* Recommended > 4 h per day	*Primary outcome:* Coronal Cobb angle *Secondary outcome:* coronal and sagittal balance, rib hump (millimetres) *Number of F/U:* 3 *Length of F/U*: T1 = 6 months T2 = 2 years T3 = ≥5 years	*Main finding:* Cobb angle increased by > 5° in 5 patients, remained stable in 15 patients and decreased in 12 patients *Additional findings:* Secondary outcomes remained stable in 17 patients, improved in 12 patients and worsened in 2 patients Some patients were able to cease wearing the brace due to a complete abolishment of their symptoms. *Statistical significance:* Summary statistics only *Brace compliance:* 60% of patients were still wearing the brace for an average of 5 h per day at T3. Brace wear ranged from 2 to 23 h per day. *Harms associated with bracing:* NA
Gallo (2014) [27]	*Design:* Case reports *Brace type:* Rigid TLSO *Trade name:* SBrace L *Material:* NA *Proposed MOA:* Restoring coronal or sagittal alignment according to the presenting features, tailored brace *Additional treatment:* Physiotherapy *Region:* NA	*Sample size:* 2 *Age:* Case 1 = 65, Case 2 = NA *Sex:* 2 Females *Diagnosis:* Degenerative scoliosis *Scoliosis Parameters:* NA *Co-morbid Illness:* NA *Prescribed Brace Wear:* Determined by the patient	*Primary outcome:* VAS and NPRS *Secondary outcome:* QOL and activity restriction *Number of F/U*: NA *Length of F/U:* NA	*Main finding:* Case 1: Reduction of 8/10 to 3/10 pain, 3/10 function up to 8/10 function Case 2: Reduction of 6 points in the VAS scale *Additional findings:* *Case 2:* Increased mobility noted *Statistical significance:* NA *Brace compliance:* NA *Harms associated with bracing:* NA
Weiss et al (2016) [26]	*Design:* Case report *Brace type:* Rigid TLSO *Trade Name:* Gensingen *Material:* NA *Proposed MOA:* Restoring coronal or sagittal alignment according to the presenting features, tailored brace *Additional treatment:* Scoliosis specific exercise (Schroth 20–60 min per day) [Tailored], off the shelf TLSO on occasion *Region:* Germany	*Sample size:* 1 *Age:* 37 *Sex:* Female *Diagnosis:* Late Onset idiopathic scoliosis *Scoliosis Parameters:* 56° thoracic curve and a 50° lumbar curve *Co-morbid Illness:* NA *Prescribed Brace Wear:* 3–4 h per day for three days per week	*Primary outcome:* VAS *Secondary outcome:* Self-perceived QOL and Cobb angle *Number of F/U*: 2 *Length of F/U:* T1 = 12 months T2 = 16 months	*Main finding:* Lower back pain relieved. Only felt during heavy lifting *Additional findings:* Lumbar Cobb angle reduced from 50° to 32° *Statistical significance:* NA *Brace compliance:* NA *Harms associated with bracing:* NA
De Mauroy et al (2016) [20]	*Design: Prospective cohort study* *Brace type:* Rigid TLSO (rigid lordosing bivalve polyethylene overlapping brace *Trade name:* Lyon Brace *Material:* Polyethylene *Proposed MOA:* Disk protection and a three-dimensional re-equilibration of the spine *Additional treatment:* Specific physiotherapy *Region:* France	*Sample size:* 158 *Age:* 56.08 (SD = 17.35) *Sex:* 144 females, 14 males *Diagnosis:* Adult scoliosis (lumbar or thoracolumbar) *Scoliosis Parameters:* 39.6 (SD = 16.76) *Co-morbid Illness:* NA *Prescribed Brace Wear:* 3 weeks of plaster cast, followed by 4 h of bracing each day for 6 months	*Primary outcome:* Coronal Cobb angle, coronal and sagittal balance, rib hump (millimetres) *Secondary outcome:* NA *Number of F/U*: 1 *Length of F/U:* T1 = 8.41 years (SD = 3.26) [Range 5–17 years]	*Main finding:* No significant change to coronal Cobb angle. 56% remained stable, 24% improved by > 5°, 20% worsened by > 5° *Additional findings:* No significant change to rib hump. Sagittal balance worsened on average *Statistical significance:* T-test (*p* = 0.008) *Brace compliance:* 23% were non-compliant with the prescribed brace wear hours *Harms associated with bracing:* No adverse events associated with bracing.
Polastri et al (2017) [27]	*Design:* Case report *Brace Type:* Modified off the shelf brace *Trade Name:* NA *Material:* Elastic *Proposed MOA:* Create coronal shifting force towards concavity of the curve *Additional Treatment:* NA *Region:* Italy	*Sample size:* 1 *Age:* 40 *Sex:* Female *Diagnosis:* Progressive AIS in an adult *Scoliosis Parameters:* 22° lumbar curve *Co-morbid Illness:* NA *Prescribed Brace Wear:* NA	*Primary outcome:* NPRS and QPDS *Secondary outcome:* NA *Number of F/U*: 6 *Length of F/U:* T1 *=* 1 month T2 = 5 months T3 = 7 months T4 = 9 months T5 = 12 months T6 = 24 months	*Main finding:* The NPRS reduced from 8.5 at T0 to 2.0 at T6. The QPDS reduced from 43 at T0 down to 15 at T6. *Additional findings:* NA *Statistical significance:* NA *Brace compliance:* Patient adhered to the prescribed treatment and it was well tolerated, and the patient was comfortable with the use of the orthosis. *Harms associated with bracing:* No adverse events reported.
Palazzo et al (2017) [28]	*Design:* Retrospective cohort study *Brace Type:* Rigid TLSO *Trade Name:* Vesinet *Material:* Plastic *Proposed MOA:* NA *Additional Treatment:* NA *Region:* France	*Sample size:* 38 *Age:* 61.3 (SD = 8.2) *Sex:* Female *Diagnosis:* 9 progressive idiopathic scoliosis, 29 degenerative scoliosis *Scoliosis Parameters:* 49.6° (SD = 17.7) *Co-morbid Illness:* NA *Prescribed Brace Wear:* Recommended > 6 h per day	*Primary outcome:* Coronal Cobb angle *Secondary outcome:* NA *Number of F/U*: 1 *Length of F/U:* T1 = ≥5 years	*Main finding:* The rate of curve progression reduced from 1.28° (SD = 0.79) at T0 to 0.21° (SD = 0.43) at T1 *Additional findings:* NA *Statistical significance:* Linear mixed-effects regression (*p* < 0.001) *Brace compliance:* 4/38 patients did not wear the brace for the prescribed hours each day *Harms associated with bracing:* NA
Zaina et al (2018) [21]	*Design:* Pilot Prospective Cohort Study *Brace Type:* Soft TLSO *Trade Name:* Peak Scoliosis Brace *Material:* Fabric, elastic, plastic *Proposed MOA:* designed to alleviate chronic pain secondary to scoliosis in adults *Additional Treatment:* NA *Region:* Italy	*Sample size:* 20 *Age:* 67.8 (SD = 10.5) *Sex:* Female *Diagnosis:* Idiopathic or degenerative scoliosis < 30° *Scoliosis Parameters:* 61.9° (SD = 12.6) *Co-morbid Illness:* 3 patients had osteoporosis *Prescribed Brace Wear:* 2–4 h per day	*Primary outcome:* Graphical rating scale *Secondary outcome:* Roland Morris questionnaire, Core outcome measurement index, Oswestry disability index. *Number of F/U*: 1 *Length of F/U:* T1 = 4 weeks	*Main finding:* Worst pain, back pain, and leg pain significantly improved from 7.15 to 5.85, from 6.55 to 5.25, and from 5.65 to 3.55, respectively at T1 *Additional findings:* All other measures showed statistically significant improvement at T1 except the Oswestry disability index scores which remained stable at T1 *Statistical significance:* Paired t-test (*p* < 0.05) for the primary outcome *Brace compliance:* All patients were compliant with the prescribed hours of brace wear *Harms associated with bracing:* All but one patient reported satisfaction with the brace saying they felt more supported

*Avg* Average, *F/U* Follow-up, *MOA* Mechanism of Action, *NPRS* Numerical pain rating scale, *QOL* Quality of life, Quebec Pain and Disability Scale, *SD* Standard Deviation, *T0* Baseline, T[X] = Follow-up point [X] e.g. *T3* Follow-up point 3, *TLSO* Thoracolumbosacral orthosis, *VAS* Visual analogue scale, *VPRS* Verbal pain rating scale, ° = Degrees (Cobb angle)

The diagnoses given to participants in the overall sample included primary de novo degenerative scoliosis (35 [10%]) or progressive idiopathic scoliosis (59 [17%]) or a combination of these two groups (20 [6%]). The diagnosis was not clearly specified or simply termed *adult scoliosis* in 225 (66%) participants. Most studies included female participants only [6, 21, 25–29]. Female participants made up at least 78% of the overall sample. The sex of 56 participants was not clearly defined in one study [30]. The mean age provided for participants in the cohort studies ranged from 41 to 68 years, and the age of participants in the case reports ranged from 37 to 65 years. Studies involved participants who were receiving treatment in either France [19, 20], Germany [6, 25, 29, 30], or Italy [21]. In the studies that detailed the primary curve type, the curve distributions were: thoracic 7%; double major curves 13%; lumbar/thoracolumbar curves 24%; and lumbar curves 57%. The mean curve magnitude in the cohort studies ranged from 37 to 50° (Cobb), and curve magnitude in the case reports ranged from 22 to 56° (Cobb). The median figure for the initial minimum number of hours of brace wear prescribed was 4 h per day (Interquartile range = 3.5 h). One study [27] instructed the participants to wear the brace as required, and two studies [6, 26] did not specify an initial brace wear prescription. The most commonly used material for the rigid braces was polyethylene [6, 19, 20, 29, 30]. Most braces [6, 19, 20, 25, 27, 29, 30] were designed with the stated intention of improving the physiological alignment of the spine in the coronal and/or sagittal plane.

A variety of outcomes were assessed: pain (measured using a validated region-specific questionnaire, or pain rating scale); Cobb angles; walking distance; progression to surgery; coronal/sagittal balance; magnitude of rib hump, quality of life; and social functioning. There was considerable heterogeneity between studies with respect to the timing of the final follow-up assessment. The final follow-up in the case reports occurred at 8 weeks [6], 16 months [25] and 24 months [26]. Follow-up data was collected > 7.5 months after baseline assessment in most studies. One of the cohort studies [21] however, perfromed the final follow-up at 4 weeks. Three studies [19, 20, 28] captured long-term follow-up data > 5 years after baseline assessment.

All studies that assessed pain [6, 21, 25–27, 29, 30] reported either modest or significant pain reduction after the application of the brace. There were mixed findings observed in the studies that tracked Cobb angle in response to bracing [19, 20, 28] revealing that curves either: improved modestly or significantly (> 5° [Cobb]); failed to progress; or progressed at a slower rate, compared to previous known rates of progression, after being braced. In some participants, curves progressed significantly (> 5° [Cobb]) despite being braced [19, 20]. A similar mixed pattern of responses was seen in the studies that tracked clinical outcomes e.g. sagittal balance. Significant functional improvement was noted in patients from three of the case studies [6, 26, 27]. An improvement in symptoms, to the extent that the brace was no longer required in some patients, was noted in two studies [19, 30].

With respect to harms/adverse events, participants from one study [28] reported ‘discomfort’ associated with bracing that was temporary and alleviated with brace adjustment. Only three studies [20, 26, 28] made explicit mention of data pertaining to harms/adverse events. Clinical and statistical heterogeneity combined with poor reporting quality hampered the pooling of results and the creation of standardized scores for the main outcomes addressed in this study. All of the cohort studies included in this review were based on clinical populations with no control-cohort included. For this reason, each of the six cohort studies were considered to have a high risk of bias. All case studies are classified as level four evidence and are known to be associated with a high risk of bias. A summary of the risk of bias assessment is presented in Table 2.

**Table 2:** Summary of the Risk of Bias Assessment based on the Newcastle-Ottawa Scale (NOS) for the Cohort Studies

				Selection				Comparability	Outcome			
Primary Author	Year	Study Design	Study Limitations	Q1	Q2	Q3	Q4	Q5	Q6	Q7	Q8	Risk of Bias
Weiss	2006	Prospective cohort study	Female participants only, no control cohort, no a priori sample size calculations discussed, no discussion of blinded assessment, poor brace compliance, poor reporting quality	✓	✗	✓	✓	✗	✓	✓	✓	*High*
Weiss	2009	Prospective cohort study	No control cohort, no a priori sample size calculations discussed, no discussion of brace compliance, no discussion of blinded assessment, poor reporting quality	✓	✗	✓	✓	✗	✓	✓	✓	*High*
De Mauroy	2011	Retrospective cohort study	Retrospective design, no control cohort, non-blinded assessment, ill defined/non-standardised follow-up periods, unable to account for potential confounding factors, poor reporting quality	✓	✗	✓	✓	✗	✓	✓	✓	*High*
De Mauroy	2016	Prospective cohort study	No control cohort, no a priori sample size calculations discussed, analysis restricted to a small compliant subset of the sample, non-specific diagnosis used, no discussion of blinded assessment, poor reporting quality	✓	✗	✓	✓	✗	✓	✓	✓	*High*
Palazzo	2017	Retrospective cohort study	Retrospective design, no control cohort, female participants only, no blinded assessment, unable to account for potential confounding factors	✗	✗	✓	✓	✗	✓	✓	✓	*High*
Zaina	2018	Prospective cohort study	Pilot study, no control cohort, very short-term follow-up	✗	✗	✓	✓	✗	✓	✓	✓	*High*

*Abbreviations*: *Q* Question, *ROB* Risk of Bias

Symbols: ✓ = Criteria satisfied, ✗ = Criteria not satisfied

NOS Criteria:

1) Representativeness of the intervention cohort

2) Selection of non-intervention cohort

3) Ascertainment of intervention

4) Demonstration that outcome of interest was not present at start of study

5) Comparability of cohorts on the basis of the design or analysis

6) Assessment of outcome

7) Was follow up long enough for outcomes to occur?

8) Adequacy of follow up of cohorts

## Discussion

The aim of this study was to review the literature on the efficacy of bracing for adults with scoliosis. To the authors’ knowledge, this is the first systematic review to specifically examine the influence of bracing in this subgroup of the population. The findings of this review would suggest that bracing may be effective, in the short- to medium-term, for reducing pain and improving function in some adult patients. There is insufficient evidence to support the use of bracing for other clinical outcomes e.g. Cobb angle. The proposed mechanism of action put forward by the authors of the included studies was that bracing would improve physiological spinal alignment. This type of thinking corresponds with Dubousset’s ‘cone of economy’ theory [31], and aligns with the current evidence on the consequences of aberrant coronal and sagittal balance [32, 33]. Interestingly, while improvements in Cobb angle, rib hump and sagittal/coronal balance were noted in some participants, these changes were not uniform within or across the included studies. Some participants continued to deteriorate despite the intervention. There are many possible reasons for these heterogenous outcomes. The study designs employed limit the extent to which potential confounding variables such as placebo effect and other forms of bias could be identified and/or controlled for. Furthermore, with only 389 subjects in total across the included studies, the effect size of bracing cannot be determined with any great certainty.

When interpreting the findings of this review it is important to note that degenerative change and normal ageing contribute to decreased flexibility and increased stiffness in the adult spine [34]. Spinal stiffness in particular is increased in patients with spinal deformity [35]. Thus, it is plausible that the spine of an adult with scoliosis may be significantly more resistant to the influence of external corrective forces such as those being exerted by a spinal brace/orthosis. This scenario makes the goal of restoring normal spinal alignment using an orthosis more challenging in adult populations. The positive results observed in some patients may therefore be the result of individual differences e.g. anthropometrics. It is also plausible that very small shifts created by the brace in the *x*, *y* and *z* axes are enough to alter symptomatology, but not sufficient to alter the clinical course of spinal deformity itself.

Some of the between-study differences may be explained by the fact that several different orthoses were used in the included studies. In the scoping review activities for this study, there were 65 terms identified relating to either brand names or generic names for different brace designs. Only six different brands of braces and one unknown (two soft and four rigid) were represented in this study. At this point in time, there are no studies that have compared the efficacy of different brace types in adult scoliosis patients. It is plausible that different approaches to bracing may produce differing outcomes. However, this line of enquiry should naturally be restrained until more robust evidence can be produced regarding the efficacy of bracing in general for adults with this condition.

Hours of brace-wear may also be an important factor in adults with scoliosis. The number of hours of brace-wear reported amongst participants in the included studies ranged from 2 to 23 h per day (median brace-wear prescription = 4 h, range of brace-wear prescribed = 2–20 h). It is known, in adolescent populations at least, that bracing outcomes are strongly influenced by compliance to brace-wear prescriptions [36, 37]. There were no objective compliance monitors (e.g. temperature sensors) used in any of the studies included in this review. Obviously, factors such as curve magnitude, flexibility, and growth potential underpin brace-wear prescriptions in skeletally immature patients, but a specific dose-response in adults may also exist. Currently there are no data from which to derive recommendations regarding this aspect of treatment in adults. In this review, five of the cohort studies [19–21, 28, 29] discussed compliance. At the final follow-up, adherence to brace wear prescriptions ranged from 24 to 100%. The reasons for non-compliance with brace-wear prescriptions may be quite varied. In some instances, poor compliance may indicate treatment success, i.e. the patient no longer feels the need to use the brace, while other cases of poor compliance may point to poor tolerance of the treatment itself or unwanted side-effects. Weiss et al [29] stated that spinal braces/orthoses place the patient’s trunk in a fixed position which can impair movement and restrict certain postures. This may not be appreciated by certain patients and decisions regarding whether to wear a brace as prescribed are likely based on an evaluation of the burdensomeness versus the perceived benefit. Weiss et al [30] stated that brace wear prescriptions in earlier studies were left up to patient preference and were not successful. This prompted researchers to modify their brace-wear prescription to a minimum of 20 h per day for the first six months. After that point, it was hypothesized that mobilization of the spine would occur which would allow for the desired functional improvements in the spine. Patients could then manage their brace-free intervals from then on.

There are limitations associated with this review. Firstly, the review protocol was not published on the international prospective register of systematic reviews (PROSPERO database). Only studies published in English were included in this review. The authors acknowledge that valuable findings from research reports published in other languages may have been omitted in this review. There is a general paucity of data on the effects of spinal bracing/orthosis treatment of adult patients with primary de novo degenerative scoliosis or progressive idiopathic scoliosis. Moreover, the data that does exist comes from a few centers in Europe, and utilizes only a handful of different brace designs, which could impact on the external validity of the findings from this review.

The majority of participants in the included studies were female. It is not clear whether the review findings can be extrapolated to adult males with scoliosis. *Adult scoliosis* was the stated diagnosis in the majority (66%) of participants included in this review. Different subtypes of adult scoliosis may respond differently to bracing, however this cannot be determined with any great certainty based on the current literature. Furthermore, head to head comparisons between brace types, and brace manufacturers were not possible in this review due to the limited research in this field and the clinical and statistical heterogeneity of the available literature.

Although not specifically addressed in this review, reporting quality in the included studies was poor overall. This also placed significant limitations on the type of analyses that could be performed. Moreover, the risk of bias assessment highlighted that the studies included in this review have a *high* risk of bias, which negatively impacts upon the internal validity and hence the certainty of the findings from these studies. Case studies have obvious methodological limitations which translates into a high risk of bias in these types of study designs. The majority of cohort studies included in this review were based on the clinical outcomes of participants receiving brace treatment for scoliosis. The usefulness of findings from cohort studies that lack a control-cohort is questionable where the exposure-outcome association is being assessed. In this type of design, there is no way of determining how the *exposed* cohort compares with a similar *non-exposed* cohort in terms of outcomes. Some studies with relative short follow-up times have been included in this review. Given the size of the literature on spinal brace/orthosis treatment for adults with scoliosis, the authors decided to summarise all the available studies and associated outcomes, acknowledging that the derivation of more precise and robust estimates of treatment effect and duration/timing of such effects will only be possible when better quality studies become available.

Despite the importance of this type of research, a review of all the international clinical trial registries highlights that our knowledge on bracing for adult scoliosis is unlikely to change in the near future due to a lack of planned research in this area. Twenty international trial registries were searched using the terms *scoliosis* or *spinal deformity.* Of the 867 results retrieved, only three of these pertained to bracing in adult populations. One trial [21] has been completed and was included in this review, and recruitment is ongoing in the remaining two studies (ClinicalTrials.gov Identifier: NCT03332277 and NCT03572855).

There is a clear requirement for high-quality research into spinal brace/orthosis treatment for adults with scoliosis. Randomised controlled trials involving a direct comparison between braced and non-braced participants would provide the most robust findings in this regard, however blinding of participants would be difficult. If cohort studies are to be used to better understand the influence of bracing in adult scoliosis they should: be prospective in nature; include an adequate and representative sample consisting of both cases and matched controls; have clearly defined diagnoses; utilize standardized patient-centered clinical and radiographic outcomes; and assess these outcomes at short, medium and long term follow-up points. The authors should also make use of appropriate guideline documents for the reporting of study findings where available e.g. STROBE statement [38].

## Conclusion

There is evidence to suggest that spinal brace/orthosis treatment may have a positive short – medium term influence on pain and function in adults with either progressive primary (de novo) degenerative scoliosis or progressive idiopathic scoliosis. At this point in time the evidence is of low quality and relates predominantly to female patients with thoracolumbar and lumbar curves, and has been based on samples drawn predominantly from Europe. More granular statements regarding the efficacy of different brace types or manufacturers, or the effect of this therapy has on different curve types cannot be determined based on the current literature. Properly constructed prospective trials are clearly required to better understand the efficacy of bracing in adult scoliosis.

## Data Availability

The datasets used and/or analysed during the current study are available from the corresponding author on reasonable request.

## Appendix

### Search String for the PubMed database

*(((artbrace OR boston brace OR milwaukee brace OR cervicothoracolumbosacral orthosis OR charleston bending brace OR charleston bending brace OR cheneau brace OR cmcr brace OR corset monocoque carbone respectat la respiration OR ctls OR derotation brace OR dynamic derotation brace OR elastic brace OR gensingen brace OR long brace OR low profile brace OR LSO OR lumbosacral orthosis OR lyon brace OR milwaukee brace OR night over-correcting brace OR night time brace OR nyrc smart brace OR ober scoliosis brace OR soliman kallabis harness OR orthosis OR over corrective brace OR pasb OR peak brace OR progressive action short brace OR providence brace OR rigid brace OR rigo-cheneau brace OR rosenberger brace OR schol brace OR scoliosis activity suit OR scoliosis brace OR schol smart activity suit OR sforzesco brace OR short brace OR short detorsional brace OR sibilla brace OR soft brace OR spinecor OR sport brace OR super rigid brace OR suspension brace OR thoracolumbar lordotic intervention brace OR thoracolumbosacral orthosis OR three point scoliosis brace OR tli brace OR tlso OR triac OR tripoint scoliosis brace OR wcr brace OR wilmington brace OR wood cheneau brace OR wood cheneau rigo brace))) AND ((scoliosis OR degenerative lumbar scoliosis OR* de novo *scoliosis OR adult spinal deformity OR degenerative scoliosis OR primary degenerative scoliosis OR progressive idiopathic scoliosis OR secondary degenerative scoliosis OR spinal deformity))) AND ((adult OR geriatric OR elderly OR aged OR aged, 80 OR over OR young adult OR middle aged))*.

## Abbreviations

LAT: Lateral
LSO: Lumbosacral orthosis
NOS: Newcastle-Ottawa Scale
PA: Posteroanterior
PDS: Primary (de novo) degenerative scoliosis
QOL: Quality of life
SOSORT: Society on Scoliosis Orthopaedic and Rehabilitation Treatment
TLSO: Thoracolumbosacral orthosis
